# Genetic characterization of a Marek’s disease virus strain isolated in Japan

**DOI:** 10.1186/s12985-020-01456-1

**Published:** 2020-11-23

**Authors:** Shiro Murata, Yuka Machida, Masayoshi Isezaki, Naoya Maekawa, Tomohiro Okagawa, Satoru Konnai, Kazuhiko Ohashi

**Affiliations:** 1 Department of Disease Control, Faculty of Veterinary Medicine, Hokkaido University, Kita-18, Nishi-9, Kita-ku, Sapporo, 060-0818 Japan; 2 Department of Advanced Pharmaceutics, Faculty of Veterinary Medicine, Hokkaido University, Kita-18, Nishi-9, Kita-ku, Sapporo, 060-0818 Japan

**Keywords:** Marek’s disease virus, Marek’s disease, Meq, Japanese strain, Whole genome sequencing

## Abstract

**Background:**

Marek’s disease virus (MDV) causes malignant lymphomas in chickens (Marek’s disease, MD). MD is currently controlled by vaccination; however, MDV strains have a tendency to develop increased virulence. Distinct diversity and point mutations are present in the Meq proteins, the oncoproteins of MDV, suggesting that changes in protein function induced by amino acid substitutions might affect MDV virulence. We previously reported that recent MDV isolates in Japan display distinct mutations in Meq proteins from those observed in traditional MDV isolates in Japan, but similar to those in MDV strains isolated from other countries.

**Methods:**

To further investigate the genetic characteristics in Japanese field strains, we sequenced the whole genome of an MDV strain that was successfully isolated from a chicken with MD in Japan. A phylogenetic analysis of the *meq* gene was also performed.

**Results:**

Phylogenetic analysis revealed that the Meq proteins in most of the Japanese isolates were similar to those of Chinese and European strains, and the genomic sequence of the Japanese strain was classified into the Eurasian cluster. Comparison of coding region sequences among the Japanese strain and MDV strains from other countries revealed that the genetic characteristics of the Japanese strain were similar to those of Chinese and European strains.

**Conclusions:**

The MDV strains distributed in Asian and European countries including Japan seem to be genetically closer to each other than to MDV strains from North America. These findings indicate that the genetic diversities of MDV strains that emerged may have been dependent on the different vaccination-based control approaches.

## Background

Marek’s disease virus (MDV; family, Herpesviridae; subfamily, α-Herpesvirinae; genus, *Mardivirus*; species, *Gallid alphaherpesvirus* 2) is the causative agent of Marek’s disease (MD), which manifests as malignant lymphomas in infected chickens [1]. MD previously caused serious economic losses to the poultry industry, but the introduction of vaccines has led to its successful control [2]. Attenuated strains of MDV and the naturally non-oncogenic *Gallid alphaherpesvirus* 3 and *Meleagrid alphaherpesvirus* 1 (turkey herpesvirus, HVT) have been used as monovalent or multivalent vaccines. An attenuated MDV strain, CVI988, is considered the most protective vaccine currently available and has been introduced in many countries [3]. However, the virulence of MDV field strains has a tendency to increase, and pathogenic MDV strains are generally classified as mild, virulent, very virulent, and very virulent+ [4]. Currently, sporadic occurrences of MD are still reported in some countries [4–11], and highly virulent MDV strains could potentially cause future outbreaks despite vaccination [4].

The Meq protein, which is highly expressed in MDV-transformed cell lines and tumor samples [12–15], is an oncoprotein of MDV. Its structure consists of an N-terminal basic region leucine zipper (bZIP) domain and a C-terminal transactivation domain. The bZIP domain, similar to that of the Jun/Fos family of oncoproteins, consists of two stretches of basic residues (basic regions 1 and 2) and a leucine zipper, whereas the transactivation domain is characterized by proline-rich repeats that contain several SH3-binding motifs [16]. Meq can form dimers via the leucine zipper and interact with the promoter region of target genes through the basic region, thereby regulating gene expression in host cells and MDV [16]. In addition, Meq can interact with p53 [17] and the C-terminal binding protein [18], ultimately affecting characteristics of transformation such as anti-apoptotic effects and regulation of gene expression. Thus, Meq plays important roles in transformation induced by MDV and is essential for viral pathogenesis.

Genetic approaches have revealed polymorphisms in Meq proteins among MDV strains isolated in USA and that they are associated with MDV virulence [19]. Amino acid substitutions can affect Meq protein functions, transactivation activities, and transformation [20, 21]. The evolution of *meq* genes is comparable with the evolutionary rate of RNA viruses, and the time of *meq* gene divergence is related to the transitions in management practices in the poultry industry, including the introduction of vaccines [23]. Thus, the positive selection induced by vaccination seems to be a reason for the emergence of genetic diversity in the *meq* gene [23]. To date, *meq* gene sequences in several regions other than the USA, including China, Europe and Australia, have been investigated [5, 24–31]. The *meq* genes in MDV strains isolated in each region were found to exhibit different genetic characteristics [23]. Thus, *meq* gene polymorphisms or evolution seem to reflect the geographical characteristics and the history of vaccine use in each region.

Whole-genome sequences of MDV strains isolated in some regions have been reported from the 2000s. The attenuated MDV vaccine strain, CVI988 [32], MDV strains with different virulence in the USA [33–38], pathogenic and vaccine strains in China [39–44], and a pathogenic strain in Europe [45, 46] were sequenced. Phylogenetic analysis indicated that most MDV strains can be classified into two clusters, namely, the North American or Eurasian clusters, and the estimated time-scaled phylogeny suggested that MDV virulence evolved independently in Eurasia and North America [46]. CVI988 was initially isolated and developed in Europe as a vaccine and is used worldwide. However, in the USA, HVT was initially used for the prevention of MD, and a bivalent vaccine comprising HVT and SB-1 (naturally non-oncogenic *Gallid alphaherpesvirus* 3) has been adopted after outbreaks in HVT-vaccinated chickens. Therefore, the history of vaccine control seems to affect the differential evolution of MDV strains in each continent [46].

In Japan, MD occurrences are sporadic. We previously reported the sequences of *meq* genes in MDV isolates in Japan and that the amino acid sequence at position 176 is serine or threonine in the most recent field isolates [22]. This amino acid sequence was found to be unique to Japanese isolates, and the sequences of *meq* are genetically closer to those of Chinese isolates [22]. To identify the properties of MDV strains distributed in Japan, we previously isolated a field strain of MDV from chickens that showed clinical signs of MD. The isolated strain Kgs-c1 was not contaminated by the three types of vaccine strains [47]. However, the genetic characteristics of Kgs-c1, except for the *meq* gene, is still unknown. Therefore, in this study, to investigate the genetic characteristics of Kgs-c1, we performed whole-genome sequencing. We also compared the sequences with those of MDV strains isolated in other countries.

## Methods

### Virus

An MDV strain, Kgs-c1, was isolated in 2014 from a poultry farm in Japan, in which some chickens had developed signs of MD [47]. Day-old-chicks from the same farm were vaccinated with CVI988, an attenuated GaHV-2 vaccine strain [47]. PCR analysis, which targeted the *meq* gene to detect CVI988 and specific genes to detect other vaccine strains and chicken anemia virus, revealed that Kgs-c1 was not contaminated with vaccine strains and chicken anemia virus (data not shown). Kgs-c1 was passaged 12 times in chicken embryo fibroblasts (CEFs) to obtain sufficient amounts of the virus genome for analysis.

### DNA preparation

Total cellular DNA samples were extracted from CEFs infected with Kgs-c1 as previously described [48]. In brief, the infected CEFs were immersed overnight at 55 °C in 1 ml of lysis buffer (0.5% SDS, 0.1 M NaCl, 10 mM tris pH 8.0, 1 mM EDTA) containing proteinase K at a final concentration of 200 μg/ml. Total cellular DNA was extracted with phenol–chloroform-isoamyl alcohol (25:24:1), precipitated with ethanol, and treated with RNase A at a final concentration of 20 μg/ml.

### Whole-genome sequencing of Kgs-c1

The concentration of total cellular DNA was determined using a Qubit Fluorometer (Invitrogen, CA, USA) and Agilent 220 TapeStation System. The total cellular DNA was used to create a library, by using the TruSeq ChIP Sample Prep Kit (Illumina K.K.). Library preparations were sequenced with an Illumina HiSeq with 100-base paired-end reads. All the kits described were used according to the manufacturers’ instructions. All procedures were performed by Hokkaido System Science Co. Ltd, Hokkaido, Japan.

### Read mapping to a reference sequence and mutation analysis

The paired-end read output from HiSeq was subjected to adaptor-trimming using cutadapt (version 1.1) (http://code.google.com/p/cutadapt/) and quality-trimmed using Trimmomatic (version 0.32) (http://www.usadellab.org/cms/?page=trimmomatic). The resulting reads were mapped to a reference sequence from RB-1B, which is a well-characterized virulent strain of MDV (accession number: EF523390) [37, 49–51] (accession number: EF523390), using Burrows-Wheeler Aligner (version 0.7.10) (http://bio-bwa.sourceforge.net/), and the output alignment data were sorted using SAMtools V (version 1.2) (http://www.htslib.org/man/samtools/) and the Genome Analysis Toolkit (Lite version 2.3.0) (https://www.broadinstitute.org.gatk/). The differences in nucleotide sequences between the Kgs-c1 and RB-1B genomes were identified using SAMtools V (version 1.2) and BCFtools (version 1.2) (https://www.htslib.org/man/bcftools/), and the consensus sequence of Kgs-c1 (accession number: LC589272) was determined based on the results of read-mapping and comparison with the RB-1B genome. The allele frequencies of the mapped reads were higher than 99% in all of the open reading frames from the consensus sequence. All the above-mentioned procedures were performed by Hokkaido System Science Co. Ltd.

### Genetic analysis of the meq and UL36 genes and the MDV genomes

Phylogenetic analysis of the *meq* gene was performed using MDV isolates from several countries including Japan, USA, China, other Asian countries, Europe, and Australia. The sequences were aligned using MAFFT version 7 [52], and the tree was constructed with MEGA7 software [53], using the minimum evolution algorithm [54]. Information about the *meq* genes used for the analysis is provided in Additional file 1: Table S1. Unique long (UL) to unique short (US) (UL–internal repeat long (IRL)–internal repeat short–UL) sequences from 26 MDV strains were aligned using MAFFT, and the tree was generated using the minimum evolution algorithm of MEGA 7 software. The *UL36* genes and the variable regions of the deduced amino acid sequences of the UL36 proteins were aligned using MAFFT. A phylogenetic tree based on *UL36* genes from 26 MDV strains, which were used for the phylogenetic analysis of UL–US sequences, was generated using the minimum evolution algorithm of MEGA 7 software. The MDV strains and their accession numbers used for this analysis are listed in Table 1.

**Table 1:** MDV strains used for the analyses in this study

Country	Strain	Virulence	Genome size (bp)	Accession No	References
Japan	Kgs-c1	–	174,999	LC589272	This study
USA	648a	vv +	176,080	JQ806361	Spatz et al. [36]
	Md5	vv	177,874	NC_002229	Tulman et al. [38]
	Md11	vv	170,950	AY510475	Niikura et al. [34]
	RB-1B	vv	178,246	EF523390	Spatz et al. [37]
	GA	v	174,077	AF147806	Lee et al. [33]
	CU-2	m	176,922	EU499381	Spatz et al. [35]
China	GX0101	vv	178,101	JX844666	Su et al. [42]
	LMS	vv	177,526	JQ314003	Cheng et al. [39]
	814	m/vaccine	172,541	JF742597	Zhang et al. [44]
	CC/1409	–	175,561	KU744560	Lv et al. [41]
	HNGS101	–	175,888	MG432697	He et al. [40]
	HNLC503	–	178,195	MG518371	–
	HS/1412	–	175,532	KU744561	Lv et al. [41]
	J-1	–	176,118	KU744555	Lv et al. [41]
	JL/1404	–	176,083	KU744559	Lv et al. [41]
	LCC	–	175,525	KU744556	Lv et al. [41]
	LCY	–	175,319	KX290013	Zhang et al. [43]
	LTS	–	176,023	KU744557	Lv et al. [41]
	WC/1203	–	176,057	KU744558	Lv et al. [41]
Poland	Polen5	hv**	177,821	MF431496	Trimpert et al. [46]
Israel	EU-1	hv	177,828	MF431494	Trimpert et al. [46]
Hungary	ATE2539	vv+	177,868	MF431493	Trimpert et al. [46]
England	pC12:130	vv	183,850*	FJ436096	Spatz et al. [45]
Hungary	MD70/13	v	177,844	MF431495	Trimpert et al. [46]
Netherland	CVI988	m/vaccine	178,311	DQ530348	Spatz et al. [32]

^*^The length of pC12:130 genome contains a vector sequence

^**^Hyper virulent (hv): Recent field strains isolated in Europe with a history of high virulence but not pathotyped within m–vv+ grading scheme [46]

## Results

### Genome organization of the Japanese strain Kgs-c1 and comparison of genome sequences between Kgs-c1 and RB-1B

The whole-genome sequence of Kgs-c1 was analyzed, and the obtained reads were mapped to the genome of the RB-1B strain, a well-characterized vv MDV strain [37, 49–51]. The consensus sequence was determined, and the length of the viral genome was estimated to be 174,999 bp. Non-synonymous differences were found in several genes between Kgs-c1 and RB-1B. Except for MDV049 (*UL36*), multi-polymorphisms were found in MDV005 (*meq*), MDV084 (*ICP4*), MDV095 (*glycoprotein I*), and MDV096 (*glycoprotein E*) (Table 2). In most cases, the differences found based on comparison with the RB-1B genome were similar to those found in the sequences of some strains from Europe and China, whereas they were different from those found in most of US strains. In contrast, the polymorphisms found near the junction between repeat long and UL sequences were unique to Kgs-c1 (MDV006–019; Table 2). An amino acid deletion was found in MDV013 (*glycoprotein L*)/MDV013.5 (*MHC class II beta chain binding protein*) (Table 3). A four-amino acid deletion was found in MDV013/MDV013.5, and this deletion was not observed in MDV013/013.5 of other strains. In MDV056 (*probable membrane protein*), the amino acid residues at positions 136 and 137 of RB-1B are asparagine and methionine, respectively, whereas the amino acid sequence in Kgs-c1 included lysine at position 136 and an amino acid was deleted. However, MDV056 sequences in Chinese and European strains were the same as those in Kgs-c1 (Table 3).

**Table 2:** Non-synonymous differences in the amino acid sequences in the coding regions between Kgs-c1 and other MDV strains

Gene name	Product	Position	JPN	USA					CHN			EU			
			Kgs-c1	RB-1B	648a	Md5	GA	CU-2	GX 0101	LMS	814	ATE 2539	C12: 130	MD70/13	CVI988
MDV004	23 kDa nuclear protein	76	*S*	R	*S*	*S*	R	R	*S*	*S*	R	R	R	R	R
MDV005	R-LORF7 (Meq)	77	*E*	K	K	K	K	*E*	*E*	*E*	*E*	*E*	*E*	K	*E*
		80	*Y*	D	D	D	D	D	*Y*	*Y*	D	*Y*	*Y*	D	D
		115	*A*	V	V	V	V	V	*A*	*A*	*A*	V	V	V	V
		176/235*	*S*	P	A	P	P	P	R	R	P	P	P	P	P
		217/ 276*	*A*	P	*A*	*A*	P	P	*A*	*A*	P	P	P	P	P
MDV006.6	B68 (VZV transducing protein)	14	*I*	T	T	T	T	T	T	T	T	T	T	T	T
MDV010	v-lipase	352	*A*	V	V	V	V	V	V	V	V	*A*	V	V	V
MDV015	UL3 (nuclear phosphoprotein)	6	*C*	G	G	G	G	G	G	G	G	G	G	P	G
MDV016	UL4 (nuclear protein)	244	*I*	V	V	V	V	V	V	V	V	V	V	V	V
MDV018	UL6 (DNA packaging, minor capsid protein)	243	*D*	N	N	N	A	N	N	N	N	N	N	N	N
MDV019	UL7 (DNA packaging/cleavage, capsid protein)	172	*K*	Q	Q	Q	Q	Q	Q	Q	Q	Q	Q	Q	Q
MDV020	UL8 (DNAhelicase-primase associated protein)	84	*D*	E	*D*	*D*	*D*	*D*	*D*	*D*	*D*	*D*	*D*	*D*	*D*
MDV020.5	UL8.5 (origin binding protein)	42	*R*	G	G	G	G	G	*R*	*R*	G	G	*R*	G	G
		462	*A*	V	V	V	V	V	V	V	V	V	V	V	V
MDV021	UL9 (origin binding protein)	395	*R*	G	G	G	G	G	*R*	*R*	G	G	*R*	G	G
		815	*A*	V	V	V	V	V	V	V	V	V	V	V	V
MDV040	UL27 (glycoprotein B)	537	*F*	S	S	S	S	S	S	S	S	S	S	S	S
MDV042	UL29 (single-strand DNA binding protein)	918	*A*	V	V	V	*A*	*A*	*A*	*A*	*A*	*A*	*A*	V	*A*
		1,183	*A*	V	V	V	*A*	*A*	*A*	*A*	*A*	*A*	*A*	V	*A*
MDV043	UL30 (DNA polymerase)	1,087	*D*	N	N	N	N	N	N	N	*D*	N	N	N	N
		1,095	*A*	V	V	V	V	V	V	V	*A*	V	V	V	V
MDV044	UL31 (nuclear phosphoprotein)	39	*R*	G	G	G	G	G	*R*	*R*	*R*	G	*R*	G	G
MDV046	UL32 (DNA packaging)	415	*T*	I	I	I	I	I	*T*	*T*	*T*	*T*	*T*	I	I
MDV047	UL34 (membrane phosphoprotein)	228	*A*	G	G	G	G	G	G	G	G	G	G	L	G
MDV049**	UL36 (large tegument protein)	3,241	*E*	K	*E*	*E*	*E*	*E*	*E*	*E*	*E*	*E*	*E*	*E*	*E*
MDV050	UL37 (tegument protein)	3	*A*	V	V	*A*	A	*A*	*A*	*A*	*A*	*A*	*A*	V	*A*
		258	*R*	Q	Q	Q	Q	Q	*R*	*R*	*R*	*R*	*R*	Q	Q
		521	*H*	R	R	R	R	R	*H*	*H*	*H*	*H*	*H*	R	R
MDV051	UL38 (capsid protein)	68	*A*	T	T	T	T	T	*A*	*A*	*A*	*A*	*A*	T	*A*
MDV055	UL42 (DNA polymerase)	114	*I*	V	V	V	V	V	*I*	*I*	*I*	*I*	*I*	V	V
		166	*V*	A	A	A	A	A	A	A	A	*V*	A	A	A
MDV056	UL43 (probable membrane protein)	305	*E*	K	K	K	*E*	K	*E*	*E*	*E*	T	*E*	K	*E*
		399	*I*	V	*I*	V	V	V	V	V	V	V	V	V	V
MDV071	LORF10 (VZV ORF2)	34	*L*	W	W	W	*L*	*L*	*L*	*L*	*L*	W	*L*	W	*L*
MDV073	R-LORF14a (pp38)	109	*G*	E	E	E	E	*G*	*G*	*G*	*G*	*G*	*G*	*G*	*G*
		115	*L*	P	P	P	P	P	P	P	P	P	P	P	P
MDV084	ICP4	181	*S*	P	*S*	P	P	P	P	P	P	P	P	P	P
		532	*R*	C	C	C	*R*	C	*R*	*R*	*R*	*R*	*R*	C	C
		1,084	*L*	P	P	P	*L*	*L*	*L*	*L*	*L*	*L*	S	P	*L*
		1,993	*P*	L	L	L	L	L	*P*	*P*	*P*	*P*	*P*	L	L
		2,017	*E*	K	K	K	K	K	*E*	*E*	*E*	*E*	*E*	K	K
		2,111	*R*	L	L	L	*R*	P	*R*	*R*	*R*	*R*	*R*	L	L
MDV091	US2 (transmission associated protein)	51	*A*	V	V	V	*A*	V	*A*	V	*A*	*A*	*A*	V	*A*
MDV092	US3 (serine/threonine protein kinase)	4	*T*	S	S	S	*T*	S	*T*	S	*T*	S	*T*	S	T
		109	*P*	S	S	S	*P*	*P*	*P*	*P*	*P*	*P*	*P*	S	*P*
MDV095	US7 (glycoprotein I)	3	*V*	L	L	L	*V*	L	*V*	L	*V*	L	Y	L	L
		112	*G*	H	H	H	*G*	H	*G*	H	*G*	D	*G*	D	H
		142	*S*	A	A	A	*S*	A	*S*	A	*S*	A	*S*	A	A
		155	*V*	A	A	A	*V*	A	*V*	A	*V*	A	*V*	A	A
		223	*V*	I	I	I	*V*	I	*V*	I	*V*	I	*V*	I	I
MDV096	US8 (glycoprotein E)	67	*I*	V	V	V	V	V	V	V	V	V	V	V	V
		370	*I*	V	V	V	V	V	V	V	*I*	*I*	V	V	V
		392	*L*	I	I	I	I	I	*L*	I	*L*	*L*	*L*	I	I
		455	*A*	D	D	D	D	D	*A*	D	*A*	*A*	*A*	T	D
		469	*D*	A	A	A	A	A	*D*	A	*D*	*D*	*D*	A	A

Italics indicated that the sequences are indentical to those of Kgs-c1

Differences in the coding regions whose functions, including the prediction, were indicated. The differences in the coding regions in Internal Repeat Long (IRL) and Terminal Repeat Short (TRS) between Kgs-c1 and RB-1B were omitted, because the same differences were contained in the same regions in Terminal Repeat Long (TRL) and Internal Repeat Short (IRS), respectively

^*^The *meq* genes in CU-2, 814 and CVI988 contain the insertion in the proline-rich repeat (PRR) sequences in the transactivation domain, and therefore, the number of PRRs are increased. The amino acid residues at positions 176 and 235 or 217 and 276 were located at PRR, and those sequences are the same

^**^Differences in MDV049 (UL36 (large tegument protein)) were indicated except for variable regions at the C-terminus

**Table 3:** In-frame deletion and insertion in the coding regions identified in Kgs-c1 and other MDV strains

Gene name	Product	JPN	USA					CHN			EU			
		Kgs-c1	RB-1B	648a	Md5	GA	CU-2	GX 0101	LMS	814	ATE 2539	C12: 130	MD70/13	CVI 988
MDV013	UL1 (glycoprotein L)	*T18-N21 (del.)*	–	–	–	–	–	–	–	–	–	–	–	–
MDV013.5	LORF4 (MHC class II beta chain binding protein)	*V125-L128 (del.)*	–	–	–	–	–	–	–	–	–	–	–	–
MDV056	UL43 (probable membrane protein)	*N136-M137 (del.)*	–	–	–	–	–	*N136-M137 (del.)*	*N136-M137 (del.)*	*N136-M137 (del.)*	*N136-M137 (del.)*	*N136-M137 (del.)*	–	–
MDV056	UL43 (probable membrane protein)	*K136 (ins.)*	–	–	–	–	–	*K136 (ins.)*	*K136 (ins.)*	*K136 (ins.)*	*K136 (ins.)*	*K136 (ins.)*	–	–

Italics indicated that the sequences are indentical to those of Kgs-c1

### Phylogenetic analysis of the sequences from UL to US regions

**Fig. 1:**
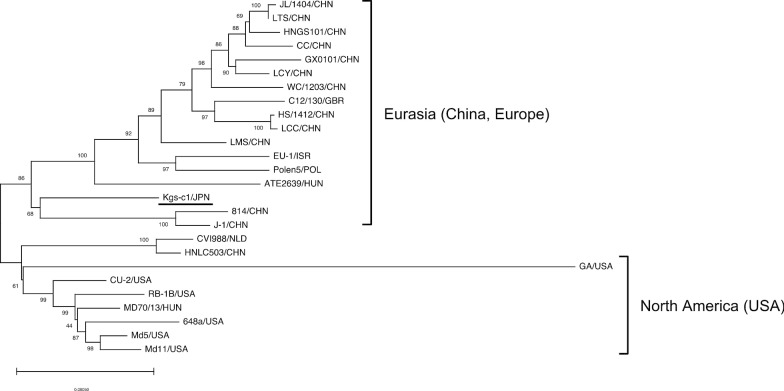
The phylogenetic tree based on sequences from UL to US. The tree of UL–US regions from Kgs-c1 and MDV strains isolated in other countries was generated based on the minimum evolution algorithm with a bootstrap analysis of 1000 replicates. The MDV genomes were classified into two clusters, Eurasia and North America. The scale indicates divergence time

The sequence of the Kgs-c1 genome, except for the terminal repeat long and terminal repeat short regions, was subjected to phylogenetic analysis. In this study, because we used a large number of nucleotides for phylogenetic analysis, the enormous computational time required is a challenge, especially for the maximum likelihood method [55]. Therefore, to analyze the genetic characteristics of the Kgs-c1 genome, we employed methods based on the minimum evolution principle [54]. The MDV strains formed two clusters, the Eurasian and North American clusters (Fig. 1). The genome of Kgs-c1 was classified into the Eurasian cluster, and these results were consistent with the results obtained by comparing the coding regions among MDV strains (Tables 2 and 3). Therefore, the ancestor of Kgs-c1 might be closer to the Chinese and European strains than to the US strains. In the Eurasian cluster, however, Kgs-c1 seemed to belong to a different branch from pathogenic strains of China and Europe, although the number of available sequences was limited.

### Phylogenetic analysis of the meq genes

**Fig. 2:**
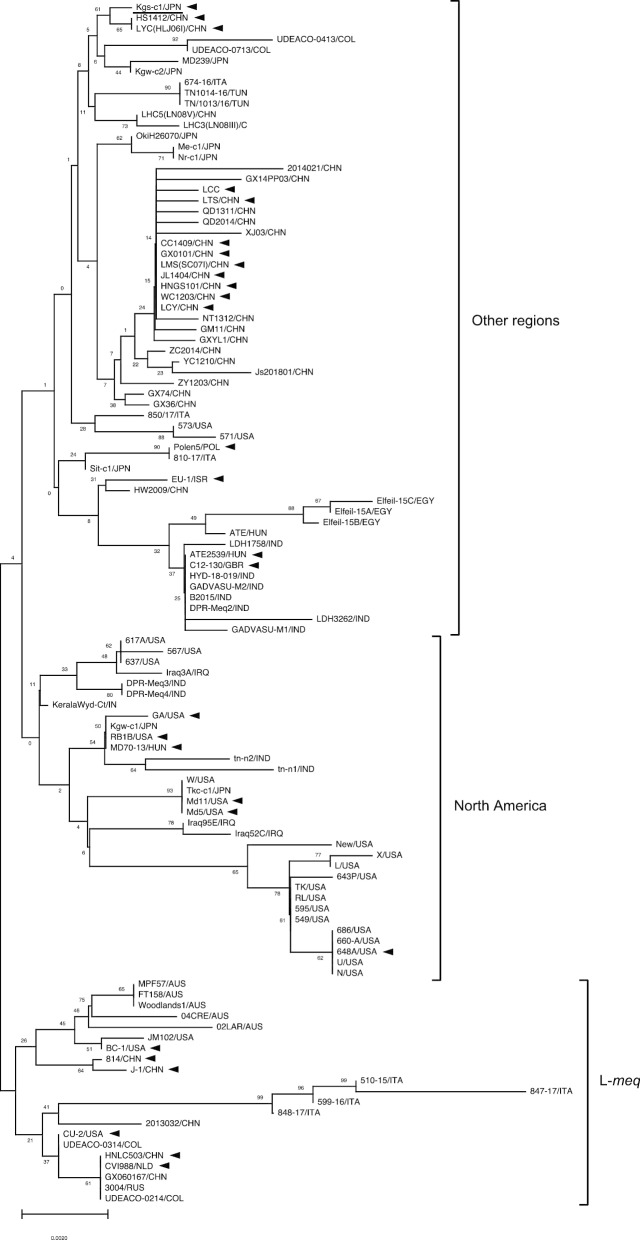
Phylogenetic trees based on alignment of *meq* genes from Kgs-c1 and MDV strains. The tree was generated using the minimum evolutionary algorithm with a bootstrap analysis of 1000 replicates. The *meq* genes used in this analysis were mainly classified into three clusters, North America, other regions including Eurasian countries, and L-*meq*. Numbers indicate bootstrap percentages (1000 replicates). Arrow heads indicate MDV strains used for the phylogenetic analysis described in Fig. 1. The scale indicates divergence time

The *meq* genes from MDV strains isolated from various countries of North America, Europe, Asia, Africa, South America, and Oceania formed three major clusters as follows: North America, other regions including Europe, Asia, Africa, and South America as well as L-*meq* (these *meq* genes contained insertions in the transactivation domains; Fig. 2). The sequence of the *meq* gene in Kgs-c1 was most frequently observed in MDV isolates in Japan [22], and therefore, most *meq* genes from Japanese isolates belonged to the other regions’ cluster. However, the *meq* genes from some isolates in Europe and Asia were classified into the North American cluster. Thus, some MDV strains, which harbor *meq,* indicative of similar genetic characteristics to those of US isolates, seem to be present in other countries as well. Strains of low virulence and pathogenic strains from Australia and Italy were mainly classified to the third cluster, L-*meq*. Geographical features and the correlation with virulence were not found in the cluster, because an insertion could largely affect the formation of the cluster.

### Comparison of the UL36 genes

**Fig. 3:**
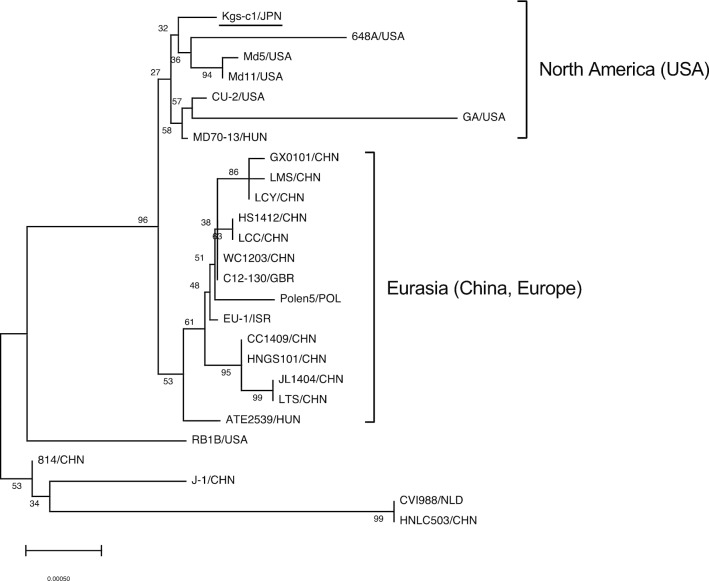
Phylogenetic trees based on alignment of the *UL36* genes from Kgs-c1 and MDV strains. The tree was generated using the minimum evolutionary algorithm with a bootstrap analysis of 1000 replicates. The *UL36* genes from MDV strains used in the analysis described in Fig. 1 were compared. The *UL36* genes used in this analysis were mainly classified into two clusters, North America and Eurasia. Numbers indicate bootstrap percentages (1000 replicates). The scale indicates divergence time

When we compared the sequences of the coding regions of MDV strains, the variable regions in the UL36 proteins were the most diverse (Additional file 2: Fig. S1). As a different feature of MDV UL36 proteins from those of other herpesviruses, the variable region is present near the C-terminus and its sequences are different among MDV strains [56]. The variable regions in the UL36 proteins of MDV consisted of two types of repeat sequences, KP(T/S/P)PA(S/P) and KPKPPP(D/A/T)PD (F/S) (Additional file 3: Table S2, Additional file 2: Fig. S1). When we compared the sequences of the variable regions among MDV strains, no trend in the number of repeat sequences was observed, correlating with the virulence (Additional file 3: Table S2). When we analyzed the phylogeny of the *UL36* genes from the same MDV strains used for the analysis of the UL–US regions (Fig. 1), we found that the sequences of the *UL36* genes were classified into two clusters, North America and Eurasia (Fig. 3). Unlike the *meq* genes from isolates of the UL–US regions, the *UL36* gene from Kgs-c1 belonged to the North American cluster, suggesting that Kgs-c1 partially has genetic characteristics different from MDV strains isolated in Eurasian countries.

## Discussion

MD is currently well-controlled by vaccination, although it previously caused serious economic losses to the poultry industry. However, the virulence and genetic characteristics of MDV strains have changed over time, and divergence seems to be correlated with the introduction of vaccines [23, 46]. According to a previous report, the genome sequences of MVD strains are classified into two main clusters, Eurasia and North America [46]. The genome sequences of MDV strains isolated in USA, China, and Europe have been investigated [32–46]. However, data on the whole genome sequences of MDV strains from other regions are limited. We previously reported changes in the genetic characteristics of *meq* genes in Japanese isolates [22]. In the present study, to compare the genetic characteristics of the whole genome of MDV strains circulating in Japan, we analyzed the whole genome sequence of a Japanese field strain, Kgs-c1, isolated in 2014.

The distinct diversity of the *meq* gene has been considered to be correlated with the enhanced virulence of MDV strains [19, 23]. The *meq* gene is thought to be associated with the evolution of MDV virulence, and non-synonymous mutations are frequently observed in *meq* genes among MDV strains [46]. To date, sequences of the *meq* genes from MDV strains from various countries have been reported [5, 24–31]. In the present study, the UL–US regions of MDV strains were divided into two clusters, Eurasia and North America, as previously reported [46], whereas the *meq* genes of MDV strains from various regions formed three clusters, North America, other regions including Eurasian countries, and L-*meq*. Unfortunately, most of the whole genome sequences of MDV strains for which *meq* genes were classified into the L-*meq* cluster, in the phylogenetic analysis of the *meq* gene, were not available. However, the L-*meq* genes of strains 814 and CU-2, which were classified into Eurasia and North America groups, respectively, possess an insertion in the transactivation domain, and therefore, these L-*meq* genes seemed to be classified into the L-*meq* cluster in this phylogenetic analysis. Thus, the *meq* gene and UL–US region seem to indicate similar phylogeny, except for the appearance of the L-*meq* cluster. The *meq* gene of Kgs-c1, for which the sequence is frequent among Japanese MDV strains, was classified into the cluster of other regions. In addition, the UL–US region of Kgs-c1 was classified into the Eurasian cluster. Taken together, the genetic characteristics of MDV strains distributed in Japan might be largely classified into the Eurasian cluster, similar to that observed with Kgs-c1. However, some *meq* genes from Japanese isolates were classified into the North American cluster. Therefore, it is possible that MDV strains with genetic characteristics similar to those of US strains are also present in Japan.

A deletion of amino acid sequences was found in MDV013 (*glycoprotein L*)/MDV013.5 (*MHC class II beta chain binding protein*) and MDV056 (*probable membrane protein*). The deletion in MDV013/MDV013.5 was also found in some highly virulent strains [19]. However, this deletion does not seem to be correlated with increased virulence [57, 58]. The Chinese and European strains showed a deletion in the same region of MDV056 [32, 39–46]. However, this deletion was also found in the vaccine strain 814 and therefore, it might not affect virulence, although its correlation with MD pathogenesis is unclear.

The historical background related to the introduction of vaccines in each country is different, and this seems to be correlated with differences in the evolution of MDV genomes in each country [46]. In Japan, HVT was initially approved for protection from MD in 1972. A few years after the initial introduction of HVT, field outbreaks were sporadically observed in HVT-vaccinated chickens. Therefore, other types of vaccines, specifically CVI988 in 1985 and a bivalent vaccine comprising CVI988 and HVT in 1988, were approved. Thereafter, CVI988 and multivalent vaccines including CVI988 have been widely used to prevent MD occurrences in poultry farms in Japan. In Europe, HPRS-16, which is an MDV strain that was originally isolated in the UK, was initially used as a live-attenuated vaccine [2]. Later, a vaccine derived from an attenuated MDV strain, CVI988, was used; currently, this vaccine is being used globally [2]. In contrast, in the USA, HVT was initially developed as a live vaccine [2]. In addition, a naturally non-pathogenic strain, SB-1, was isolated in the USA in the late 1970s, and has been used as a bivalent vaccine to enhance the vaccine efficacy of HVT [2]. The historical background related to the use of vaccines between Europe and the USA is thus different, and the background of Japan is closer to that of Europe. Thus, the use of vaccines could induce the evolution of MDV strains in each country, and Japanese strains seemed to develop genetic characteristics similar to those of European strains.

The UL36 protein, a large tegument protein encoded by a member of Herpesviridae, is known to form the innermost layer of the complex protein scaffold between the capsid and envelope [59]. MDV encodes a ubiquitin-specific protease as part of the N-terminal region of the UL36 protein, similar to that observed in other known herpesviruses [60]. The UL36 protein was found to be correlated with the tumorigenic activity and replication of MDV via the deubiquitinase activity of the ubiquitin-specific protease [60, 61]. In contrast, MDV encodes unique repeat sequences at the C-terminal region [56]. Phylogenetic analysis revealed that the *UL36* gene of Kgs-c1 was classified into the North American cluster, unlike those of the *meq* gene and UL–US region. In addition, Kgs-c1 exhibited unique sequences in the coding regions at the 5′ regions of the UL region. Thus, Kgs-c1 might have undergone evolutionary processes different from those of other pathogenic strains, although Kgs-c1 is closer to Chinese and European strains than to US strains in terms of the whole genome sequence.

For the phylogenetic analysis, methods based on the maximum likelihood principle have been often applied, as their accuracy is generally higher than that of other methods. However, these methods require much longer time, and some factors, such as the number of nucleotides, the number of sequences, and the model used for analysis, often affect accuracy [55]. Therefore, when using these methods, optimization should be considered. In addition, many empirical studies have indicated that other methods, including the minimum evolution principle, provide similar phylogenetic inference by applying the bootstrap test [55]. A previous study reported that in metagenomic analysis, phylogenetic classification using the novel method (*PhyClass*) based on the minimum-evolution principle was as efficient as that with the maximum likelihood methods [62]. Therefore, in the present study, the genetic characteristics of the Kgs-c1 genome were analyzed using the minimum-evolution method. However, its accuracy might be less than those using the best existing maximum likelihood methods with optimized models. Therefore, other approaches should be applied to analyze the phylogeny of Kgs-c1 more accurately, and assess the biases by the minimum-evolution method.

## Conclusion

In this study, we investigated the genomic characteristics of a Japanese MDV strain, namely Kgs-c1, by analyzing its whole genome sequence. The genomic features of Kgs-c1 were largely related to those of Chinese and European strains, rather than US strains. In Japan, CVI988 has been widely used to prevent MD occurrences, and therefore, this study supports the hypotheses that the historical background of vaccine use affects the evolution of MDV strains and that MDV strains adapt to the immunity induced by vaccination to be distributed in the field. However, the genome sequence of Kgs-c1 partially indicates unique characteristics in some coding regions. Thus, the ancestor of MDV strains distributed in Japan, China, and Europe seems to be closer to each other than to strains from the USA, but the evolution of Japanese strains might be unique.

## Supplementary information

**Additional file 1: Table S1.** The *meq* genes used for analyses in this study.

**Additional file 2: Fig. S1.** Alignment of the deduced amino acid sequences of the variable regions of UL36 proteins. The variable regions in the UL36 proteins from Kgs-c1 and MDV strains isolated in other countries were aligned. Shaded fields in gray indicate the repeat sequence of “KP (T/S/P)PA(S/P)”, and a solid line indicates each repeat sequence. The different colors of the letters indicate each type of the repeat sequence of “KP (T/S/P)PA(S/P)”. Patterned fields indicate the repeat sequence of “KPKPPP(D/A/T)PD(F/S)”, and a dotted line indicates each repeat sequence. The number of each repeat sequence is summarized in Additional file 1: Table S1.

**Additional file 3: Table S2.** Number of repeat sequences in the UL36 proteins.

## Data Availability

The nucleotide sequence of the Kgs-c1 genome is accessible in GeneBank under accession number LC589272.

## Abbreviations

MDV: Marek’s disease virus
MD: Marek’s disease
HVT: Turkey herpesvirus
bZIP: Basic leucine zipper
CEF: Chicken embryo fibroblast
UL: Unique long
US: Unique short
IRL: Internal repeat long
