# Supplementary material for: Genetic characterization of a Marek’s disease virus strain isolated in Japan
Source: Virol J. 2020 Nov 23;17:186. doi: 10.1186/s12985-020-01456-1 (PMC7684920; doi:10.1186/s12985-020-01456-1)
Supplement: Supplementary file 1 — Additional file 1: Table S1. The meq genes used for analyses in this study. [file 12985_2020_1456_MOESM1_ESM.pdf]

Supplementary Table 1. The *meq* genes used for the analyses in this study

	strain	country	accession No.	year (collection)	year (submission)	authors
1	CVI988	NLD	AY243337.1	-	2003	Shamblin,C.E., Greene,N., Arumugaswami,V., Dienglewicz,R.L. and Parcells,M.S.
2	GXYL1	CHN	HQ290415.1	2005	2010	Wei,P. and Zhou,X.
3	MD/HYD/18/019	IND	MK388089.1	2018	2019	Kannaki,T.R., Danutha,N.R., Priyanka,E. and Radhika,P.
4	LYC(HLJ/06/I)	CHN	HQ658627.1	2006	2010	Zhang,Y. and Liu,C.
5	LMS(SC/07/I)	CHN	HQ658622.1	2007	2010	Zhang,Y. and Liu,C.
6	LHC5(LN/08/V)	CHN	HQ658619.1	2008	2010	Zhang,Y. and Liu,C.
7	LHC3(LN/08/III)	CHN	HQ658617.1	2008	2010	Zhang,Y. and Liu,C.
8	GaHV-2/Italy/Ck/848/17	ITA	MK139673.1	2017	2018	Mescolini,G., Lupini,C., Felice,V., Guerrini,A., Silveira,F., Cecchinato,M. and Catelli,E.
9	GaHV-2/Italy/Ck/847/17	ITA	MK139672.1	2017	2018	Mescolini,G., Lupini,C., Felice,V., Guerrini,A., Silveira,F., Cecchinato,M. and Catelli,E.
10	GaHV-2/Italy/Ck/810/17	ITA	MK139671.1	2017	2018	Mescolini,G., Lupini,C., Felice,V., Guerrini,A., Silveira,F., Cecchinato,M. and Catelli,E.
11	GaHV-2/Italy/Ck/674/16	ITA	MK139667.1	2016	2018	Mescolini,G., Lupini,C., Felice,V., Guerrini,A., Silveira,F., Cecchinato,M. and Catelli,E.
12	GaHV-2/Italy/Ck/599/16	ITA	MK139665.1	2016	2018	Mescolini,G., Lupini,C., Felice,V., Guerrini,A., Silveira,F., Cecchinato,M. and Catelli,E.
13	GaHV-2/Italy/Ck/510/15	ITA	MK139663.1	2015	2018	Mescolini,G., Lupini,C., Felice,V., Guerrini,A., Silveira,F., Cecchinato,M. and Catelli,E.
14	TN1014/16	TUN	KY113150.1	2016	2016	Lachheb,J., Mastour,H., Turki,A., Kaboudi,K., Nsiri,J., Elbehi,I. and Ghram,A.
15	ATE	HUN	AY571784.1	-	2004	Dren,C.N., Kumar,P.M. and Parcells,M.S.
16	KeralaWyd-Ct	IND	MK584548.1	2014	2019	Priya,M.P. and Mangottumuruppel,M.
17	Elfeil-15C	EGY	MH428673.1	2015	2018	Ifeil,W.K., Abouelmaatti,R.R. and Elfeil,L.
18	Elfeil-15B	EGY	MH428672.1	2015	2018	Ifeil,W.K., Abouelmaatti,R.R. and Elfeil,L.
19	Elfeil-15A	EGY	MH428671.1	2015	2018	Ifeil,W.K., Abouelmaatti,R.R. and Elfeil,L.
20	Js201801	CHN	MK046676.1	2018	2018	Bai,X., Wu,Y. and Zhang,X.
21	GADVASU-M2	IND	KY651232.1	2016	2017	Varte,L., Singh,A., Deka,D., Singh,S., Sharma,D., Gupta,K. and Verma,R.
22	GADVASU-M1	IND	KY651231.1	2016	2017	Varte,L., Singh,A., Deka,D., Singh,S., Sharma,D., Gupta,K. and Verma,R.
23	B2015	IND	LC195187.1	2015	2016	Puro,K.U. and Bhattacharjee,U.
24	DPR-Meq3	IND	KT795531.1	2014	2015	Balena,V. and Reddy,M.R.
25	DPR-Meq2	IND	KT795530.1	2014	2015	Balena,V. and Reddy,M.R.
26	ZC2014	CHN	KP144356.1	2014	2014	Zhang,K., Gong,Z., Lin,X., Wang,H., Zhang,L., Hou,G., Yu,J. and Shan,H.
27	QD2014	CHN	KP144354.1	2014	2014	Zhang,K., Gong,Z., Li,L., Lin,X., Zhang,Q., Zhang,L., Yu,J. and Shan,H.
28	tn-n2	IND	HM749325.1	2010	2010	Sathish,G., Kurunchi Divya,C., Parthiban,M. and Kumanan,K.
29	tn-n1	IND	HM749324.1	2010	2010	Sathish,G., Kurunchi Divya,C., Parthiban,M. and Kumanan,K.
30	UDEACO-04/13	COL	KU058701.1	2014	2015	Lopez-Osorio,S., Espinal-Restrepo,M.A., Piedrahita,D., Ramirez-Nieto,G.C., Nair,V., Williams,S.M., Baigent,S.J. and Chaparro-Gutierrez,J.J.
31	UDEACO-03/14	COL	KU058699.1	2014	2015	Lopez-Osorio,S., Espinal-Restrepo,M.A., Piedrahita,D., Ramirez-Nieto,G.C., Nair,V., Williams,S.M., Baigent,S.J. and Chaparro-Gutierrez,J.J.
32	UDEACO-02/14	COL	KU058698.1	2014	2015	Lopez-Osorio,S., Espinal-Restrepo,M.A., Piedrahita,D., Ramirez-Nieto,G.C., Nair,V., Williams,S.M., Baigent,S.J. and Chaparro-Gutierrez,J.J.
33	UDEACO-07/13	COL	KU058697.1	2013	2015	Lopez-Osorio,S., Espinal-Restrepo,M.A., Piedrahita,D., Ramirez-Nieto,G.C., Nair,V., Williams,S.M., Baigent,S.J. and Chaparro-Gutierrez,J.J.
34	2014021	CHN	KU382455.1	2014	2016	Han,C., Niu,X., Peng,W. and Zeng,X.
35	2013032	CHN	KU382454.1	2013	2016	Han,C., Niu,X., Peng,W. and Zeng,X.
36	QD/1311	CHN	KP888856.1	2013	2015	Zhang,Y.
37	NT/1312	CHN	KP888855.1	2013	2015	Zhang,Y.
38	ZY/1203	CHN	KP888842.1	2012	2015	Zhang,Y.
39	YC/1210	CHN	KP888840.1	2012	2015	Zhang,Y.
40	WC/1203	CHN	KP888839.1	2012	2015	Zhang,Y.
41	LTS	CHN	KP888838.1	2012	2015	Zhang,Y.
42	GM/11	CHN	KP888825.1	2011	2015	Zhang,Y.
43	LCC	CHN	KP888815.1	2011	2015	Zhang,Y.
44	HW/2009	CHN	KP888814.1	2009	2015	Zhang,Y.
45	XJ03	CHN	HQ638155.1	-	2010	Tian,M. and Huang,Y.
46	J-1	CHN	HQ190957.1	-	2010	Tian,M.X., Huang,Y. and Deng,R.
47	GX060167	CHN	EU697887.1	2006	2008	Wei,P. and Teng,L.
48	GX14PP03	CHN	KX506775.1	2014	2016	Wei,P., Wang,P.K., Zou,G.Z., Yang,Y.L. and Lin,L.L.
49	GX74	CHN	KT229641.1	2014	2015	Qu,S.J., Shi,K.C., Mo,S.L., Zou,L.B., Hu,J. and Yin,Y.W.
50	GX36	CHN	KT229640.1	2013	2015	Qu,S.J., Shi,K.C., Mo,S.L., Zou,L.B., Hu,J. and Yin,Y.W.
51	3004 (vaccine)	RUS	EU032468.1	-	2007	Shulpin,M.I., Kozlov,A.I., Scherbakova,L.O., Kulashbekova,S.K. and Drygin,V.V.
52	RB1B	USA	HM488349.1	-	2010	Kumar,P.M., Dong,H., Lenihan,D., Katneni,U., Shaikh,S., Gaddamanugu,S., Tavlarides-Hontz,P., Reddy,S. and Parcells,M.S.
53	JM102	USA	HM488348.1	-	2010	Kumar,P.M., Dong,H., Lenihan,D., Katneni,U., Shaikh,S., Gaddamanugu,S., Tavlarides-Hontz,P., Reddy,S. and Parcells,M.S.
54	686	USA	AY362727.1	-	2003	Shamblin,C.E., Greene,N.M., Arumugaswami,V., Dienglewicz,R.L. and Parcells,M.S.
55	660-A	USA	AY362726.1	-	2003	Shamblin,C.E., Greene,N.M., Arumugaswami,V., Dienglewicz,R.L. and Parcells,M.S.
56	648A	USA	AY362725.1	-	2003	Shamblin,C.E., Greene,N.M., Arumugaswami,V., Dienglewicz,R.L. and Parcells,M.S.
57	X	USA	AY362724.1	-	2003	Shamblin,C.E., Greene,N.M., Arumugaswami,V., Dienglewicz,R.L. and Parcells,M.S.
58	W	USA	AY362723.1	-	2003	Shamblin,C.E., Greene,N.M., Arumugaswami,V., Dienglewicz,R.L. and Parcells,M.S.
59	U	USA	AY362722.1	-	2003	Shamblin,C.E., Greene,N.M., Arumugaswami,V., Dienglewicz,R.L. and Parcells,M.S.
60	TK	USA	AY362721.1	-	2003	Shamblin,C.E., Greene,N.M., Arumugaswami,V., Dienglewicz,R.L. and Parcells,M.S.
61	RL	USA	AY362720.1	-	2003	Shamblin,C.E., Greene,N.M., Arumugaswami,V., Dienglewicz,R.L. and Parcells,M.S.
62	New	USA	AY362719.1	-	2003	Shamblin,C.E., Greene,N.M., Arumugaswami,V., Dienglewicz,R.L. and Parcells,M.S.
63	N	USA	AY362718.1	-	2003	Shamblin,C.E., Greene,N.M., Arumugaswami,V., Dienglewicz,R.L. and Parcells,M.S.
64	L	USA	AY362717.1	-	2003	Shamblin,C.E., Greene,N.M., Arumugaswami,V., Dienglewicz,R.L. and Parcells,M.S.
65	643P	USA	AY362716.1		2003	Shamblin,C.E., Greene,N.M., Arumugaswami,V., Dienglewicz,R.L. and Parcells,M.S.
66	595	USA	AY362715.1		2003	Shamblin,C.E., Greene,N.M., Arumugaswami,V., Dienglewicz,R.L. and Parcells,M.S.
67	549	USA	AY362714.1		2003	Shamblin,C.E., Greene,N.M., Arumugaswami,V., Dienglewicz,R.L. and Parcells,M.S.
68	637	USA	AY362713.1		2003	Shamblin,C.E., Greene,N.M., Arumugaswami,V., Dienglewicz,R.L. and Parcells,M.S.
69	617A	USA	AY362712.1		2003	Shamblin,C.E., Greene,N.M., Arumugaswami,V., Dienglewicz,R.L. and Parcells,M.S.
70	573	USA	AY362711.1		2003	Shamblin,C.E., Greene,N.M., Arumugaswami,V., Dienglewicz,R.L. and Parcells,M.S.
71	571	USA	AY362710.1		2003	Shamblin,C.E., Greene,N.M., Arumugaswami,V., Dienglewicz,R.L. and Parcells,M.S.
72	567	USA	AY362709.1		2003	Shamblin,C.E., Greene,N.M., Arumugaswami,V., Dienglewicz,R.L. and Parcells,M.S.
73	CU-2	USA	AY362708.1		2003	Shamblin,C.E., Greene,N.M., Arumugaswami,V., Dienglewicz,R.L. and Parcells,M.S.
74	BC-1	USA	AY362707.1		2003	Shamblin,C.E., Greene,N.M., Arumugaswami,V., Dienglewicz,R.L. and Parcells,M.S.
75	Woodlands1	AUS	EF523775.1		2007	Renz,K.G., Cheetham,B.F. and Walkden-Brown,S.W.
76	MPF57	AUS	EF523774.1		2007	Renz,K.G., Cheetham,B.F. and Walkden-Brown,S.W.
77	04CRE	AUS	EF523773.1		2007	Renz,K.G., Cheetham,B.F. and Walkden-Brown,S.W.

78	02LAR	AUS	EF523772.1		2007	Renz,K.G., Cheetham,B.F. and Walkden-Brown,S.W.
79	FT158	AUS	EF523771.1		2007	Renz,K.G., Cheetham,B.F. and Walkden-Brown,S.W.
80	Kgw-c2	JPN	LC385874.1	2012	2018	Murata,S. and Ohashi,K.
81	MD239	JPN	LC385873.1	1980	2018	Murata,S. and Ohashi,K.
82	Me-c1	JPN	LC385872.1	2004	2018	Murata,S. and Ohashi,K.
83	Nr-c1	JPN	LC385871.1	2004	2018	Murata,S. and Ohashi,K.
84	Sit-c1	JPN	LC385870.1	2016	2018	Murata,S. and Ohashi,K.
85	Tkc-c1	JPN	LC385869.1	2004	2018	Murata,S. and Ohashi,K.
86	Kgw-c1	JPN	LC385868.1	2010	2018	Murata,S. and Ohashi,K.
87	DPR-Meq4	IND	KX619428.1	2014	2016	Balena,V. and Reddy,M.R.
88	MDV-OkiH26070-2014	JPN	LC137001.1	2014	2016	Nidaira,M. and Matsukawa,K.
89	LDH-3262	IND	KF895035.1	2012	2013	Gupta,M., Deka,D. and Verma,R.
90	LDH-1758	IND	KF895029.1	2012	2013	Gupta,M., Deka,D. and Verma,R.
91	Iraq95E	IRQ	KC243270.1	2010	2012	Wajid,S.J.
92	Iraq52C	IRQ	KC243267.1	2010	2012	Wajid,S.J.
93	Iraq3A	IRQ	KC243262.1	2010	2012	Wajid,S.J.
94	TN 1013/16	TUN	MK041219.1	2016	2018	Lachheb,J. and Nsiri,J.
95	GaHV-2/Italy/Ck/850/17	ITA	MK139674.1	2017	2018	Mescolini,G., Lupini,C., Felice,V., Guerrini,A., Silveira,F., Cecchinato,M. and Catelli,E.
96	Kgs-c1	JPN	Shown in Table 1			
97	814	CHN	Shown in Table 1			
98	CC/1409	CHN	Shown in Table 1			
99	ATE2539	HUN	Shown in Table 1			
100	EU-1	ISR	Shown in Table 1			
101	GA	USA	Shown in Table 1			
102	GX0101	CHN	Shown in Table 1			
103	HNGS0101	CHN	Shown in Table 1			
104	HNLC503	CHN	Shown in Table 1			
105	HS/1412	CHN	Shown in Table 1			
106	JL/1404	CHN	Shown in Table 1			
107	LCY	CHN	Shown in Table 1			
108	Md5	USA	Shown in Table 1			
109	Md11	USA	Shown in Table 1			
110	MD70/13	HUN	Shown in Table 1			
111	C12:130	GBR	Shown in Table 1			
112	Polen5	POL	Shown in Table 1			
