# Supplementary material for: Genetic characterization of a Marek’s disease virus strain isolated in Japan
Source: Virol J. 2020 Nov 23;17:186. doi: 10.1186/s12985-020-01456-1 (PMC7684920; doi:10.1186/s12985-020-01456-1)
Supplement: Supplementary file 3 — Additional file 3: Table S2. Number of repeat sequences in the UL36 proteins. [file 12985_2020_1456_MOESM3_ESM.docx]

|  | | | | | | | | | | | |
| --- | --- | --- | --- | --- | --- | --- | --- | --- | --- | --- | --- |
| **Supplementary table 2.** Number of repeat sequences in the UL36 proteins | | | | | | | | | | | |
| Country | Strain | Virulence | KP(T/S/P)PA(S/P) | | | | | | | KPKPPP(D/A/T)PD(F/S) | |
|  |  |  | Total | KPTPAS | KPSPAS | KPTPAP | KPSPAP | KPPPAS | KPPPAP |  |  |
| Japan | Kgs-c1 | - | 16 | 0 | 4 | 6 | 6 | 0 | 0 | 6 | |
| USA | 648a | vv+ | 20 | 0 | 7 | 7 | 6 | 0 | 0 | 6 | |
|  | Md5 | vv | 21 | 0 | 4 | 11 | 6 | 0 | 0 | 7 | |
|  | Md11 | vv | 18 | 0 | 4 | 8 | 6 | 0 | 0 | 7 | |
|  | RB1B | vv | 20 | 0 | 5 | 7 | 8 | 0 | 0 | 9 | |
|  | GA | v | 18 | 0 | 5 | 6 | 6 | 0 | 1 | 7 | |
|  | CU-2 | m | 17 | 0 | 5 | 5 | 6 | 0 | 1 | 7 | |
| China | GX0101 | vv | 20 | 1 | 7 | 6 | 5 | 0 | 1 | 8 | |
|  | LMS | vv | 19 | 0 | 7 | 6 | 5 | 0 | 1 | 7 | |
|  | 814 | m/vaccine | 1 | 0 | 0 | 1 | 0 | 0 | 0 | 0 | |
|  | CC/1409 | - | 20 | 0 | 8 | 6 | 5 | 0 | 1 | 7 | |
|  | HNGS101 | - | 20 | 0 | 8 | 6 | 5 | 0 | 1 | 4 | |
|  | HNLC503 | - | 23 | 0 | 0 | 4 | 16 | 1 | 2 | 5 | |
|  | HS/1412 | - | 16 | 0 | 6 | 6 | 3 | 0 | 1 | 5 | |
|  | J-1 | - | 16 | 0 | 1 | 4 | 9 | 1 | 1 | 8 | |
|  | JL/1404 | - | 18 | 0 | 8 | 5 | 4 | 0 | 1 | 6 | |
|  | LCC | - | 16 | 0 | 6 | 6 | 3 | 0 | 1 | 5 | |
|  | LCY | - | 20 | 0 | 7 | 7 | 5 | 0 | 1 | 5 | |
|  | LTS | - | 17 | 0 | 8 | 5 | 3 | 0 | 1 | 5 | |
|  | WC/1203 | - | 19 | 0 | 7 | 6 | 5 | 0 | 1 | 7 | |
| Poland | Polen5 | hv | 21 | 0 | 5 | 10 | 5 | 0 | 1 | 7 | |
| Israel | EU-1 | hv | 21 | 0 | 6 | 10 | 4 | 0 | 1 | 7 | |
| Hungary | ATE2539 | vv+ | 20 | 0 | 6 | 7 | 6 | 0 | 1 | 8 | |
| England | pC12:130 | vv | 19 | 0 | 7 | 6 | 5 | 0 | 1 | 7 | |
| Hungary | MD70/13 | v | 21 | 0 | 5 | 10 | 5 | 0 | 1 | 7 | |
| Netherland | CVI988 | m/vaccine | 20 | 0 | 0 | 4 | 13 | 1 | 2 | 5 | |
| m, mild; v, virulent; vv, very virulent; vv+, very virulent +; hv, hyper virulent | | | | | | | | | | |  |
