# Supplementary material for: Genetic characterization of a Marek’s disease virus strain isolated in Japan
Source: Virol J. 2020 Nov 23;17:186. doi: 10.1186/s12985-020-01456-1 (PMC7684920; doi:10.1186/s12985-020-01456-1)
Supplement: Supplementary file 2 — Additional file 2: Fig. S1. Alignment of the deduced amino acid sequences of the variable regions of UL36 proteins. The variable regions in the UL36 proteins from Kgs-c1 and MDV strains isolated in other countries were aligned. Shaded fields in gray indicate the repeat sequence of “KP (T/S/P)PA(S/P)”, and a solid line indicates each repeat sequence. The different colors of the letters indicate each type of the repeat sequence of “KP (T/S/P)PA(S/P)”. Patterned fields indicate the repeat sequence of “KPKPPP(D/A/T)PD(F/S)”, and a dotted line indicates each repeat sequence. The number of each repeat sequence is summarized in Additional file 1: Table S1. [file 12985_2020_1456_MOESM2_ESM.pdf]

```
Kgs-c1/JPN    NLFPSDHDPTSESSSKPTPAPKPTPAPKPTPAPKPKPPPDPDFKPSPAPKPSPASKPTPAPKPSPAPKPKPPPDPDFKPTPAP------------------KPKPPPDPDFKPSPAPKPSP
648A/USA      NLFPSDHDPTSESSSKPTPAPKPTPAPKPSPAS----------KPTPAPKPSPAP------------KPKPPPDPDFKPSPAPKPSPASKPTPAPKPSPAP----------KPSPAP----
Md5/USA       NLFPSDHDPTSESSSKPTPAPKPTPAPKPTPAP----------KPTPAPKPTPAPKPTPAPKPTPAPKPKPPPDPDFKPSPAPKPSPASKPTPAPKPSPAPKPKPPPDPDFKPTPAPKPSP
Md11/USA      NLFPSDHDPTSESSSKPTPAPKPTPAPKPTPAP----------KPTPAP------------------KPKPPPDPDFKPSPAPKPSPASKPTPAPKPSPAPKPKPPPDPDFKPTPAPKPSP
RB1B/USA      NLFPSDHDPTSESSSKPTPAPKPTPAPKPTPAPKPKPPPDPDFKPSPAPKPSPASKPTPAP------KPKPPPDPDFKPTPAPKPSPAS------------KPKPPPDPDFKPTPAP----
GA/USA        NLFPSDHDPTSESSSKPTPAPKPTPAPKPTPAP----------------------------------KPKPPPDPDFKPSPAPKPSPASKPTPAPKPPPAPKPKPPPDPDFKPTPAPKPSP
CU-2/USA      NLFPSDHDPTSESSSKPTPAPKPTPAP------KPKPPPDPDFKPSPAPKPSPASKPTPAPKPPPAPKPKPPPDPDFKPTPAPKPSPAS------------KPKPPPDPDFKPTPAP----
GX0101/CHN    NLFPSDHDPTSESSSKPTPAPKPTPAPKPTPAP----------------------------------KPKPPPDPDFKPSPAPKPSPASKPTPAPKPPPAPKPKPPPDPDFKPTPAPKPSP
LMS/CHN       NLFPSDHDPTSESSSKPTPAPKPTPAPKPTPAP----------------------------------KPKPPPDPDFKPSPAPKPSPASKPTPAPKPPPAPKPKPPPDPDFKPTPAPKPSP
814/CHN       NLFPSDHDPTSESSSKPTPAP----------------------------------------------------------------------------------------------------
CC1409/CHN    NLFPSDHDPTSESSSKPTPAPKPTPAPKPTPAP----------------------------------KPKPPPDPDFKPSPAPKPSPASKPTPAPKPPPAP--------------------
HNGS101/CHN   NLFPSDHDPTSESSSKPTPAPKPTPAPKPTPAP----------------------------------KPKPPPDPDFKPSPAPKPSPASKPTPAPKPPPAPKPKPPPDPDFKPTPAPKPSP
HNLC503/CHN   NLFPSDHDPTSESSSKPTPAPKPTPAPKPPPAP----------KPPPAP------------------KPKPPPDPDFKPTPAPKPPPAS------------KPKPPPDPDFKPTPAP----
HS1412/CHN    NLFPSDHDPTSESSSKPTPAPKPTPAPKPTPAP----------KPTPAP------------------KPKPPPDPDFKPSPAPKPSPASKPTPAPKPPPAPKPKPPPDPDFKPTPAP----
J-1/CHN       NLFPSDHDPTSESSSKPTPAPKPTPAPKPPPAPKPKPPPDPDFKPTPAPKPPPAS----------------------------------------------KPKPPPAPDFKPTPAP----
JL1404/CHN    NLFPSDHDPTSESSSKPTPAPKPTPAPKPTPAP----------------------------------KPKPPPDPDFKPSPAPKPSPASKPTPAPKPPPAPKPKPPPDPDFKPTPAPKPSP
LCC/CHN       NLFPSDHDPTSESSSKPTPAPKPTPAPKPTPAP----------KPTPAP------------------KPKPPPDPDFKPSPAPKPSPASKPTPAPKPPPAPKPKPPPDPDFKPTPAP----
LCY/CHN       NLFPSDHDPTSESSSKPTPAPKPTPAPKPTPAP----------KPTPAP------------------KPKPPPDPDFKPSPAPKPSPASKPTPAPKPPPAPKPKPPPDPDFKPTPAPKPSP
LTS/CHN       NLFPSDHDPTSESSSKPTPAPKPTPAPKPTPAP----------------------------------KPKPPPDPDFKPSPAPKPSPASKPTPAPKPPPAPKPKPPPDPDFKPTPAPKPSP
WC1203/CHN    NLFPSDHDPTSESSSKPTPAPKPTPAPKPTPAP----------------------------------KPKPPPDPDFKPSPAPKPSPASKPTPAPKPPPAPKPKPPPDPDFKPTPAPKPSP
Polen5/POL    NLFPSDHDPTSESSSKPTPAPKPTPAPKPTPAP----------KPTPAPKPTPAPKPTPAPKPTPAPKPKPPPDPDFKPSPAPKPSPASKPTPAPKPPPAPKPKPPPDPDFKPTPAPKPSP
EU-1/ISR      NLFPSDHDPTSESSSKPTPAPKPTPAPKPTPAP----------KPTPAPKPTPAPKPTPAPKPTPAPKPKPPPDPDFKPSPAPKPSPASKPTPAPKPPPAPKPKPPPDPDFKPTPAPKPSP
ATE2539/HUN   NLFPSDHDPTSESSSKPTPAPKPTPAP------KPKPPPDPDFKPSPAPKPSPASKPTPAPKPTPAPKPKPPPDPDFKPSPAPKPSPASKPTPAPKPPPAPKPKPPPDPDFKPTPAPKPSP
C12-130/GBR   NLFPSDHDPTSESSSKPTPAPKPTPAPKPTPAP----------------------------------KPKPPPDPDFKPSPAPKPSPASKPTPAPKPPPAPKPKPPPDPDFKPTPAPKPSP
MD70-13/HUN   NLFPSDHDPTSESSSKPTPAPKPTPAPKPTPAP----------KPTPAPKPTPAPKPTPAPKPTPAPKPKPPPDPDFKPSPAPKPSPASKPTPAPKPPPAPKPKPPPDPDFKPTPAPKPSP
CVI988/NLD    NLFPSDHDPTSESSSKPTPAPKPTPAPKPPPAP----------KPPPAP------------------KPKPPPDPDFKPTPAPKPPPAS------------KPKPPPDPDFKPTPAP----

Kgs-c1/JPN    APKPKPPPDPDFKPTPAPKPSPASKPSPASKPSPAS-------------------------------------------------------------------------------------
648A/USA      --KPKPPPDPDFKPTPAPKPSPAS------------------------------KPKPPPDPDFKPTPAP------KPKPPPDPDFKPTPAP-----------------------------
Md5/USA       ASKPKPPPDPDFKPTPAP------------------------------------KPKPPPDPDFKPSPAPKPSPAPKPKPPPDPDFKPTPAP-----------------------------
Md11/USA      ASKPKPPPDPDFKPTPAP------------------------------------KPKPPPDPDFKPSPAPKPSPAPKPKPPPDPDFKPTPAP-----------------------------
RB1B/USA      --KPKPPPDPDFKPSPAPKPSPAP------------------------------KPKPPPDPDFKPTPAP------KPKPPPDPDFKPSPAPKPSPAP------------------KPKPP
GA/USA        ASKPKPPPDPDFKPTPAP------------------------------------KPKPPPDPDFKPSPAPKPSPAPKPKPPPDPDFKPSPAP-----------------------------
CU-2/USA      --KPKPPPDPDFKPSPAPKPSPAP----------------------------------------------------KPKPPPDPDFKPSPAP-----------------------------
GX0101/CHN    ASKPKPPPDPDFKPTPAS------------------------------------KPKPPPDPDFKPTPAP------KPKPPPDPDFKPSPAPKPSPAP------------------KPKPP
LMS/CHN       ASKPKPPPDPDFKPTPAP------------------------------------KPKPPPDPDFKPSPAPKPSPAPKPKPPPDPDFKPSPAS-----------------------------
814/CHN       -------------------------------------------------------------------------------------------------------------------------
CC1409/CHN    --KPKPPPDPDFKPTPAPKPSPAS------------------------------KPKPPPDPDFKPTPAP------KPKPPPDPDFKPSPAPKPSPAP------------------KPKPP
HNGS101/CHN   ASKPKPPPDPDFKPTPAP------------------------------------KPKPPPDPDFKPSPAPKPSPAPKPKPPPDPDFKPSPAS-----------------------------
HNLC503/CHN   --KPKPPPDPDFKPSPAPKPSPAPKPSPAPKPSPAPKPSPAPKPSPAPKPSPAP----------KPSPAPKPSPAPKPKPPPDPDFKPSPAPKPSPAPKPSPAPKPSPAPKPSPAP-----
HS1412/CHN    --KPKPPPDPDFKPSPASKPSPASKPSPASKPSPASKPSPAS-------------------------------------------------------------------------------
J-1/CHN       --KPKPPPDPDFKPSPAPKPSPAP------------------------------KPKPPPDPDFKPSPAP------KPKPPPDPDFKPSPAPKPSPAPKPSPAP------------KPKPP
JL1404/CHN    ASKPKPPPDPDFKPSPASKPSPASKPSPASKPSPASKPSPASKPSPAS------KPKPPPAPDSKPSPAP----------------------------------------------KPKPP
LCC/CHN       --KPKPPPDPDFKPSPASKPSPASKPSPASKPSPASKPSPAS-------------------------------------------------------------------------------
LCY/CHN       ASKPKPPPDPDFKPTPAP------------------------------------KPKPPPDPDFKPSPAPKPSPAPKPKPPPDPDFKPSPAS-----------------------------
LTS/CHN       ASKPKPPPDPDFKPSPASKPSPASKPSPASKPSPASKPSPASKPSPAS-------------------------------------------------------------------------
WC1203/CHN    ASKPKPPPDPDFKPTPAP------------------------------------KPKPPPDPDFKPSPAPKPSPAPKPKPPPDPDFKPSPAS-----------------------------
Polen5/POL    ASKPKPPPDPDFKPTPAP------------------------------------KPKPPPDPDFKPSPAPKPSPAPKPKPPPDPDFKPSPAS-----------------------------
EU-1/ISR      ASKPKPPPDPDFKPTPAP------------------------------------KPKPPPDPDFKPSPAPKPSPASKPKPPPDPDFKPSPAS-----------------------------
ATE2539/HUN   ASKPKPPPDPDFKPTPAP------------------------------------KPKPPPDPDFKPSPAPKPSPAPKPKPPPDPDFKPSPAS-----------------------------
C12-130/GBR   ASKPKPPPDPDFKPTPAP------------------------------------KPKPPPDPDFKPSPAPKPSPAPKPKPPPDPDFKPSPAS-----------------------------
MD70-13/HUN   ASKPKPPPDPDFKPTPAP------------------------------------KPKPPPDPDFKPSPAPKPSPAPKPKPPPDPDFKPSPAS-----------------------------
CVI988/NLD    --KPKPPPDPDFKPSPAPKPSPAPKPSPAPKPSPAPKPSPAPKPSPAPKPSPAP----------KPSPAP------KPKPPPDPDFKPSPAPKPSPAPKPSPAP-----------------

Kgs-c1/JPN    -----------------------------------------KPKPPPAPDSKPSPAPKPKPPPTPDSKPSPAPKPKSPSASKPLPVPFPNSDSKTSPVPNPNTFSASKIPPTSSIAEETKP
648A/USA      -----KPSPASKPSPASKPSPASKPSPAS------------KPKPPPAPDSKPSPAPKPKPPPTPDSKPSPAPKPKSPSASKPLPVPFPNSDSKTSPVPNPNTFSASKIPPTSSIAEETKP
Md5/USA       -----KPSPASKPSPAS------------------------KPKPPPAPDSKPSPAPKPKPPPTPDSKPSPAPKPKSPSASKPLPVPFPNSDSKTSPVPNPNTFSASKIPPTSSIAEETKP
Md11/USA      -----KPSPASKPSPAS------------------------KPKPPPAPDSKPSPAPKPKPPPTPDSKPSPAPKPKSPSASKPLPVPFPNSDSKTSPVPNPNTFSASKIPPTSSIAEETKP
RB1B/USA      PDPDFKPSPASKPSPAPKPSPASKPSPAS------------KPKPPPAPDSKPSPAPKPKPPPTPDSKPSPAPKPKSPSASKPLPVPFPNSDSKTSPVPNPNTFSASKIPPTSSIAEETKP
GA/USA        -----KPSPASKPSPASKPSPAS------------------KPKPPPAPDSKPSPAPKPKPPPTPDSKPSPAPKPKSPSASKPLPVPFPNSDSKTSPVPNPNTFSASKIPPTSSIAEETKP
CU-2/USA      -----KPSPASKPSPASKPSPAS------------------KPKPPPAPDSKPSPAPKPKPPPTPDSKPSPAPKPKSPSASKPLPVPFPNSDSKTSPVPNPNTFSASKIPPTSSIAEETKP
GX0101/CHN    PDPDFKPSPASKPSPASKPSPASKPSPASKPSPAS------KPKPPPAPDSKPSPAPKPKPPPTPDSKPSPAPKPKSPSASKPLPVPFPNSDSKTSPVPNPNTFSASKIPPTSSIAEETKP
LMS/CHN       -----KPSPASKPSPASKPSPASKPSPAS------------KPKPPPAPDSKPSPAPKPKPPPTPDSKPSPAPKPKSPSASKPLPVPFPNSDSKTSPVPNPNTFSASKIPPTSSIAEETKP
814/CHN       -------------------------------------------------------------------------KPKSPSASKPLPVPFPNSDSKTSPVPNPNTFSASKIPPTSSIAEETKP
CC1409/CHN    PDPDFKPSPASKPSPASKPSPASKPSPASKPSPASKPSPASKPKPPPAPDSKPSPAPKPKPPPTPDSKPSPAPKPKSPSASKPLPVPFPNSDSKTSPVPNPNTFSASKIPPTSSIAEETKP
HNGS101/CHN   -----KPSPASKPSPASKPSPASKPSPASKPSPAS------KPKPPPAPDSKPSPAPKPKPPPTPDSKPSPAPKPKSPSASKPLPVPFPNSDSKTSPVPNPNTFSASKIPPTSSIAEETKP
HNLC503/CHN   ---------------------------------------------------KPSPAPKPKPPPAPDSKPSPAPKPKSPSASKPLPVPFPNSDSKTSPVPNPNTFSASKIPPTSSIAEETKP
HS1412/CHN    -----------------------------------------KPKPPPAPDSKPSPAPKPKPPPTPDSKPSPAPKPKSPSASKPLPVPFPNSDSKTSPVPNPNTFSASKIPPTSSIAEETKP
J-1/CHN       PDPDFKPSPASKPSPAP------------------------KPKPPPAPDSKPSPAPKPKPPPTPDSKPSPAPKPKSPSASKPLPVPFPNSDSKTSPVPNPNTFSASKIPPTSSIAEETKP
JL1404/CHN    PDPDF----------------------------------------------KPSPAPKPKPPPTPDSKPSPAPKPKSPSASKPLPVPFPNSDSKTSPVPNPNTFSASKIPPTSSIAEETKP
LCC/CHN       -----------------------------------------KPKPPPAPDSKPSPAPKPKPPPTPDSKPSPAPKPKSPSASKPLPVPFPNSDSKTSPVPNPNTFSASKIPPTSSIAEETKP
LCY/CHN       -----KPSPASKPSPASKPSPASKPSPAS------------KPKPPPAPDSKPSPAPKPKPPPTPDSKPSPAPKPKSPSASKPLPVPFPNSDSKTSPVPNPNTFSASKIPPTSSIAEETKP
LTS/CHN       -----------------------------------------KPKPPPAPDSKPSPAPKPKPPPTPDSKPSPAPKPKSPSASKPLPVPFPNSDSKTSPVPNPNTFSASKIPPTSSIAEETKP
WC1203/CHN    -----KPSPASKPSPASKPSPASKPSPAS------------KPKPPPAPDSKPSPAPKPKPPPTPDSKPSPAPKPKSPSASKPLPVPFPNSDSKTSPVPNPNTFSASKIPPTSSIAEETKP
Polen5/POL    -----KPSPASKPSPAS------------------------KPKPPPAPDSKPSPAPKPKPPPTPDSKPSPAPKPKSPSASKPLPVPFPNSDSKTSPVPNPNTFSASKIPPTSSIAEETKP
EU-1/ISR      -----KPSPASKPSPAS------------------------KPKPPPAPDSKPSPAPKPKPPPTPDSKPSPAPKPKSPSASKPLPVPFPNSDSKTSPVPNPNTFSASKIPPTSSIAEETKP
ATE2539/HUN   -----KPSPASKPSPAS------------------------KPKPPPAPDSKPSPAPKPKPPPTPDSKPSPAPKPKSPSASKPLPVPFPNSDSKTSPVPNPNTFSASKIPPTSSIAEETKP
C12-130/GBR   -----KPSPASKPSPASKPSPASKPSPAS------------KPKPPPAPDSKPSPAPKPKPPPTPDSKPSPAPKPKSPSASKPLPVPFPNSDSKTSPVPNPNTFSASKIPPTSSIAEETKP
MD70-13/HUN   -----KPSPASKPSPAS------------------------KPKPPPAPDSKPSPAPKPKPPPTPDSKPSPAPKPKSPSASKPLPVPFPNSDSKTSPVPNPNTFSASKIPPTSSIAEETKP
CVI988/NLD    ---------------------------------------------------KPSPAPKPKPPPAPDSKPSPAPKPKSPSASKPLPVPFPNSDSKTSPVPNPNTFSASKIPPTSSIAEETKP
```
